# The expanding role of RNA modifications in plant RNA polymerase II transcripts: highlights and perspectives

**DOI:** 10.1093/jxb/erad136

**Published:** 2023-04-20

**Authors:** Marta Zimna, Jakub Dolata, Zofia Szweykowska-Kulinska, Artur Jarmolowski

**Affiliations:** Department of Gene Expression, Institute of Molecular Biology and Biotechnology, Adam Mickiewicz University, Poznan, Poland; Department of Gene Expression, Institute of Molecular Biology and Biotechnology, Adam Mickiewicz University, Poznan, Poland; Department of Gene Expression, Institute of Molecular Biology and Biotechnology, Adam Mickiewicz University, Poznan, Poland; Department of Gene Expression, Institute of Molecular Biology and Biotechnology, Adam Mickiewicz University, Poznan, Poland; Instituto de Agrobiotecnología del Litoral, Argentina

**Keywords:** Epitranscriptomics, *N* ^1^-methyladenosine, *N* ^6^-methyladenosine, 5-methylcytosine, NAD^+^ capping, RNA methylation, RNA modifications

## Abstract

Regulation of gene expression is a complicated process based on the coordination of many different pathways, including epigenetic control of chromatin state, transcription, RNA processing, export of mature transcripts to the cytoplasm, and their translation into proteins. In recent years, with the development of high-throughput sequencing techniques, the importance of RNA modifications in gene expression has added another layer to this regulatory landscape. To date, >150 different types of RNA modifications have been found. Most RNA modifications, such as *N*^6^-methyladenosine (m^6^A) and pseudouridine (**Ψ**), were initially identified in highly abundant structural RNAs, such as rRNAs, tRNAs, and small nuclear RNAs (snRNAs). Current methods provide the opportunity to identify new types of modifications and to precisely localize them not only in highly expressed RNAs but also in mRNA and small RNA molecules. The presence of modified nucleotides in protein-coding transcripts can affect their stability, localization, and further steps of pre-mRNA maturation. Finally, it may affect the quality and quantity of protein synthesis. In plants, the epitranscriptomic field is still narrow, but the number of reports is growing rapidly. This review presents highlights and perspectives of plant epitranscriptomic modifications, focusing on various aspects of modifications of RNA polymerase II transcripts and their influence on RNA fate.

## Teaser

The plant epitranscriptomic field is rapidly growing, emphasizing the role of modifications in low-abundant RNAs. We summarize current knowledge on modified ribonucleotides in RNA polymerase II transcripts and show new perspectives.

## Introduction

Chemical modifications of RNA have been known for over 50 years, with pseudouridine as the first described, also called the ‘fifth nucleotide’ (Cohn, 1960). Several years later, the first methylated mRNAs were reported in mammalian cells (Desrosiers *et al.*, 1974; Perry and Kelley, 1974) and later in plant cells (Nichols, 1979b); furthermore, capping of eukaryotic mRNAs was also described (Shatkin, 1976; Nichols, 1979a). Currently, we recognize dozens of different modification types that are present in all domains of life, in all four RNA bases as well as in the nucleoside ribose moiety (Boccaletto *et al.*, 2022). All classes of RNA can be modified starting from effector RNAs such as rRNA and tRNA through mRNAs, ending with non-coding RNAs (e.g. miRNAs, long-noncoding RNAs). The great expansion of this interesting field, now known as epitranscriptomics, in the last few years is a result of significant improvement in the methods allowing the detection of modified ribonucleotides using HPLC-MS (Thuring *et al.*, 2016), specific antibodies (Dominissini *et al.*, 2012, 2016; Meyer *et al.*, 2012; Edelheit *et al.*, 2013; Schwartz *et al.*, 2013; Luo *et al.*, 2014; Li *et al.*, 2016; Shen *et al.*, 2016; Cui *et al.*, 2017; Gu and Liang, 2019), chemical compound treatment (Carlile *et al.*, 2014, 2015; Sun *et al.*, 2019), and clickchemistry (Jiao *et al.*, 2017; Walters *et al.*, 2017; Y. Wang *et al.*, 2019; Yu *et al.*, 2021a, b), followed by next-generation sequencing (NGS) technologies, including direct RNA sequencing with the Nanopore technique (Parker *et al.*, 2020; Leger *et al.*, 2021). A growing amount of data has resulted in the development of new computational tools (reviewed in L. Liu *et al*., 2020; Furlan *et al.*, 2021), including those dedicated to plant datasets (Wang and Yan, 2018; Zhai *et al.*, 2018; Qin *et al.*, 2022). Furthermore, a number of databases collecting various epitranscriptome-related information were established, such as MODOMICS (Boccaletto *et al.*, 2022), RENAME for modification enzymes (Nie *et al.*, 2022), and many more for high-throughput data sharing and presentation (S. Liu *et al*., 2020; Ma *et al.*, 2020; Tang *et al.*, 2021).

Several high-throughput studies have been performed on plants, revealing the widespread distribution of *N*^6^-methyladenosine (m^6^A) ( Y. Li *et al*., 2014; Luo *et al.*, 2014; Shen *et al.*, 2016; Zhou *et al.*, 2019; Miao *et al.*, 2020; Parker *et al.*, 2020; Hu *et al.*, 2021; Zheng *et al.*, 2021), *N*^1^-methyladenosine (m^1^A) (Yang *et al.*, 2020), 5-methylcytosine (m^5^C) (Cui *et al.*, 2017; David *et al.*, 2017; Yang *et al.*, 2019; Tang *et al.*, 2020), and pseudouridine (Ψ) (Sun *et al.*, 2019). Moreover, other RNA modifications were identified transcriptome-wide in eukaryotic (but not plant) mRNAs, such as *N*^6^,2ʹ-*O*-dimethyladenosine (m^6^Am) (Linder *et al.*, 2015; Mauer *et al.*, 2017; Boulias *et al.*, 2019), 5-hydroxymethylcytosine (hm^5^C) (Delatte *et al.*, 2016), 2ʹ-*O*-methylated nucleosides (Nms) (Dai *et al.*, 2017), and inosine (I) (Levanon *et al.*, 2004).

It is clear, however, that some of the modifications mentioned above are much more abundant than others (e.g. m^6^A versus hm^5^C) and may play more important roles in the RNA life cycle. In most cases, the biological meaning of the modifications described still needs to be uncovered.

RNA modifications are crucial in the regulation of gene expression and have a great influence on every stage of eukaryotic mRNA function and fate. The role of modified ribonucleotides starts from the regulation of transcription through maturation (capping, splicing, and polyadenylation), ending with an influence on RNA transport and decay. Epitranscriptomic changes affect the structure of non-coding RNAs such as miRNA precursors and potentially may determine the function of deriving small RNAs (Bhat *et al.*, 2020). The importance of RNA modification in plants can be also elucidated from the severe phenotype of mutants of writer protein genes, such as *MTA* (*N*^6^-adenosine-methyltransferase MT-A70-like, a homolog of human METTL3—the m^6^A writer) (Zhong *et al.*, 2008; Bodi *et al.*, 2012) or *NAP57* (Nopp140-associated protein of 57 kDa, a homolog of human dyskerin and yeast Cbf5p—the pseudouridine writer) (Maceluch *et al.*, 2001; Kannan *et al.*, 2008). In the following sections, we will summarize current knowledge about an emerging role for modified ribonucleotides in the maturation and functioning of RNA polymerase II (RNAPII)-derived transcripts in plants.

## A role for RNA modifications in the regulation of transcription

Since the first discovery of eukaryotic RNA polymerase activity over half a century ago, transcription has been a well-studied process. The application of high-resolution cryo-EM provided detailed knowledge on the transcription complexes at different stages of RNA synthesis by RNAPII (reviewed in Hanske *et al.*, 2018). Currently, we know about the kinetics of the process and about dozens of transcription factors involved. In recent years, we have learned much about how the chromatin state regulates the synthesis of RNAs (reviewed in Cramer, 2019; Osman and Cramer, 2020). Moreover, it has been reported that non-coding RNAs may also affect the transcription of other genes. For example, in mammals, a subset of chromosome-associated regulatory RNAs (carRNAs), namely promoter-associated RNA (paRNA), enhancer RNA (eRNA), and RNA transcribed from transposable elements, are known to regulate transcription by altering chromatin architecture at corresponding genomic loci (reviewed in Li and Fu, 2019). Interestingly, it has been proven that in mice, carRNAs can be methylated by the METTL3 (methyltransferase 3) protein and that the reduction in m^6^A levels increases the stability of carRNAs, leading to better chromatin accessibility and higher transcription rates (J. Liu *et al*., 2020). It was also shown by several laboratories that m^6^A in animals can indirectly influence gene transcription by impacting epigenetic histone modifications and chromatin structure (Wang *et al.*, 2018; Li *et al.*, 2020; Xu *et al.*, 2021).

As of today, the most promising hint of RNA modifications being involved in transcription regulation in plants comes from the discovery of the role of m^6^A in R-loop structure formation (P. Zhang *et al*., 2021). R-loops consist of a DNA‒RNA heteroduplex and one displaced single strand of DNA (reviewed in Kim and Wang, 2021). R-loops are widely known to act as transcription regulators in mammals (Ginno *et al.*, 2012, 2013; Powell *et al.*, 2013; Boque-Sastre *et al.*, 2015). Studies in Arabidopsis and rice showed that R-loop structures play a role in multiple processes, including: DNA replication, transcription, alternative splicing, miRNA biogenesis, maintaining genome stability, and the regulation of flowering time as well as root development (Sun *et al.*, 2013; Conn *et al.*, 2017; Shafiq *et al.*, 2017; Z. Yang *et al.*, 2017; Fang *et al.*, 2019; Gonzalo *et al.*, 2022).

R-loops have also been studied in the context of epigenetics and epitranscriptomics. Two DNA modifications, DNA-5-methylcytosine (D-5mC) and DNA-*N*^6^-methyladenine (D-6mA), were shown to affect R-loop formation in rice (Fang *et al.*, 2019). Another study suggests that the formation and stability of R-loops and transcription of overlapping genes in plants could be enhanced by the presence of both D-6mA (a DNA modification) and R-m^6^A (an RNA modification), suggesting a possible interplay between marks on different nucleic acids (P. Zhang *et al*., 2021).

## Novel modifications of RNA polymerase II transcript 5ʹ ends

All RNAPII transcripts are characterized by a specific structure formed at their 5ʹ ends, called the 5ʹ-cap. The eukaryotic mRNA 5ʹ-end cap was first described in the 1970s (Shatkin, 1976). It is synthesized co-transcriptionally, immediately after the first 20–30 nucleotides have been transcribed (Rasmussen and Lis, 1993).

Canonical m^7^G (*N*^7^-methylguanosine) eukaryotic caps are synthesized by three enzymes (triphosphatase, guanyltransferase, and guanine-*N*^7^ methyltransferase) that subsequently modify the 5ʹ end of RNA molecules synthesized by RNAPII. All those steps result in the creation of a structure known as cap 0, which can be further modified. Methylation of the 2ʹ-*O*-ribose of the nucleotide adjacent to cap 0 results in the creation of cap 1, and the same reaction carried out on both the first and second nucleotides next to the cap creates cap 2 (reviewed in Wiedermannova *et al.*, 2021). Additionally, the hypermethylated cap, also known as the 2,2,7-trimethylguanosine (TMG) cap or the m^3^G cap, is present in most eukaryotic small nuclear ribonucleoproteins (snRNPs), as well as in some small nucleolar RNAs (snoRNAs) and mRNAs (Cheng *et al.*, 2020). This modification is known to function as a signal for U snRNPs to be transported from the cytoplasm, where these particles are assembled, to the nucleus—the site of U snRNP activity (Fischer and Luhrmann, 1990). The hypermethylated cap might also improve translation of some mammalian mRNAs encoding selenoproteins (Wurth *et al.*, 2014).

Recent years have shed new light on the 5ʹ ends of prokaryotic transcripts. A range of new, non-canonical cap-like structures have been described, including metabolic cofactors, dinucleotide analogs, and even cell wall precursors. Surprisingly, some of those unusual structures were also found in eukaryotic organisms, namely the NAD^+^/NADH cap (oxidized/reduced NAD cap), the FAD cap, and the UDP-Glc/UDP-GlcNAc cap (glycosylation cofactor UDP-glucose/UDP-*N*-acetyloglucosamine caps) (reviewed in Wiedermannova *et al.*, 2021; Mattay, 2022), but only the first has been described thus far to appear also in plant mRNAs (Y. Wang *et al.*, 2019; Zhang *et al.*, 2019; Pan *et al.*, 2020; Yu *et al.*, 2021a).

NAD^+^ has long been known as a cellular metabolite and cofactor in many reactions. Together with its reduced form, NADH, NAD^+^ is a key player in the regulation of the cellular redox state (reviewed in Gakiere *et al.*, 2018). The reduction/oxidation function of NAD makes it also crucial in plant metabolism, as the molecule acts as a coenzyme for reactions involved in biosynthesis pathways, catabolism processes, and production of energy. In human cells, the NAD^+^ caps were shown to act in an opposite manner to the canonical m^7^G caps—they promote degradation of specific transcripts (Jiao *et al.*, 2017). In animals, the enzyme responsible for decapping (‘deNADding’) and degrading NAD^+^-capped mRNAs is known as DXO (Jiao *et al.*, 2017). An *Arabidopsis thaliana* DXO homolog, the DXO1 protein, possesses both 5ʹ–3ʹ exonuclease and NAD-RNA decapping activities, similar to its animal counterpart (Jiao *et al.*, 2017; Kwasnik *et al.*, 2019; Pan *et al.*, 2020). The loss-of-function *dxo1-1* and *dxo1-2* Arabidopsis transgenic lines showed enhanced accumulation of RDR6-dependent small non-coding RNA (Kwasnik *et al.*, 2019; Pan *et al.*, 2020; Yu *et al.*, 2021a) derived from NAD^+^-capped mRNAs (Yu *et al.*, 2021b), and were probably produced alternatively to the degradation pathway. The mutants were also characterized by developmental defects (Kwasnik *et al.*, 2019; Pan *et al.*, 2020). Interestingly, the expression levels of genes involved in plant defense against pathogens (e.g. *PR1* and *PR2*) were elevated in these mutants in comparison with wild-type plants. This activation of the immune response to *dxo1* loss-of-function Arabidopsis mutants suggests an autoimmunity phenotype (Pan *et al.*, 2020). In Arabidopsis, NAD^+^-capped transcripts can be found in both nuclear and mitochondrial mRNAs, and are widespread within plant tissues (Y. Wang *et al.*, 2019). Interestingly, no transcripts from the chloroplast genome were found to possess this alternative cap structure (Y. Wang *et al.*, 2019). As the NAD^+^-capped mRNAs were found to be less stable in both wild-type Arabidopsis and the loss-of-function *dxo1* transgenic lines, it has been suggested that this specific 5ʹ-end modification can function as a destabilizing mark in the transcriptome; however, the mechanism behind this phenomenon remains undiscovered (Yu *et al.*, 2021b).

RNA modifications, specifically uridylation, also seem to be connected to the process of cap removal (decapping), as a crosstalk between decapping activator (DCP5) and UTP:RNA URIDYLTRANSFERASE 1 (URT1, protein catalyzing uridylation) has been described in Arabidopsis (Scheer *et al.*, 2021).

## The influence of RNA modifications on pre-mRNA splicing: still to be investigated in plants

Pre-mRNA splicing is carried out by the spliceosome. This macromolecule complex consists of multiple snRNPs that are responsible for the recognition of specific sequences within introns and exons (reviewed in Meyer *et al.*, 2015) These RNA‒RNA interactions may be affected by co-transcriptional RNA modification of both pre-mRNAs and small nuclear RNAs (snRNAs).

The connections between splicing and mRNA modifications (mainly m^6^A, but also pseudouridine) have been reported in various studies on animals (Zhao *et al.*, 2014; Geula *et al.*, 2015; Haussmann *et al.*, 2016; Lence *et al.*, 2016; Ke *et al.*, 2017; Mendel *et al.*, 2021; Martinez *et al.*, 2022), but to date no similar observations have been made in plant organisms. In fact, research conducted on Arabidopsis and rice mutants (with decreased expression levels of FIP37, VIR, and OsFIP proteins involved in forming plant m^6^A writer complexes) showed that splicing and alternative splicing are not significantly affected by the reduced RNA methylation level in plants (Shen *et al.*, 2016; Ruzicka *et al.*, 2017; Zhang *et al.*, 2019; Parker *et al.*, 2020).

As mentioned, chemical RNA modifications can also influence mRNA splicing indirectly, through snRNA molecules, that can be widely modified across different organisms (Chen *et al.*, 2020) (reviewed in Bohnsack and Sloan, 2018; Morais *et al.*, 2021; Ramakrishnan *et al.*, 2022). From all snRNAs involved in the action of the major spliceosome, only U6 is a product of RNAPIII transcription; the rest are synthesized by RNAPII (Kunkel *et al.*, 1986; Waibel and Filipowicz, 1990; Kiss *et al.*, 1991). Modifications such as 2ʹ-*O*-methylation, pseudouridine, and m^6^A in both RNAPII- and RNAPIII-dependent snRNAs have been suspected to play a role in snRNP–RNA interactions for a long time (reviewed in Bohnsack and Sloan, 2018; Morais *et al.*, 2021), although most of the studies are focused on animals. In plants, the strongest evidence of an interplay between modified snRNA and its role in splicing comes from U6 snRNA. Two independent studies demonstrated that the Arabidopsis FIONA1 protein, a genetic regulator of period length in the circadian clock (Kim *et al.*, 2008), also functions as a methyltransferase depositing m^6^A on plant U6 snRNA (C. L. Wang *et al*., 2022; Xu *et al.*, 2022). The proof that m^6^A modulates splicing indirectly was supplied by Parker and colleagues, when they showed that FIONA1-mediated methylation of U6 snRNA plays a role in 5ʹ splice site (5ʹSS) selection (Parker *et al.*, 2022).

## Regulation of polyadenylation of RNAPII-derived transcripts by RNA modifications

Polyadenylation can be briefly described as the cleaving of pre-mRNA at a specific site and then the addition of a tail built from non-templated adenine nucleotides (J. Yang *et al*., 2021). Both cleavage and addition of the poly(A) tail are coupled with other transcript biogenesis events (reviewed in Hunt *et al.*, 2012). It is worth mentioning that most of the primary transcripts can have multiple polyadenylation sites, meaning that many different variants of mature mRNA molecules can be produced from transcripts of one gene. This phenomenon is known as alternative polyadenylation (APA) and occurs in both animal and plant cells. In fact, it has been shown that up to 70% of Arabidopsis transcripts have more than one polyadenylation site in their primary sequence (Wu *et al.*, 2011). Many protein complexes are involved in cleavage and polyadenylation: the CPSF complex (cleavage and polyadenylation specificity factor), the CstF complex (cleavage stimulatory factor), CFI and CFII (cleavage factor I and cleavage factor II complexes, respectively), the symplekin protein, and poly(A) polymerases (PAP) (reviewed in Neve *et al.*, 2017).

Interestingly, the Arabidopsis CPSF30 gene encodes two protein variants: CPSF30-L (70 kDa longer version) and CPSF30-S (28 kDa shorter version) (Delaney *et al.*, 2006; Hou *et al.*, 2021). The longer version possesses an additional YTH domain (Delaney *et al.*, 2006), which, when present in RNA-binding proteins, is able to specifically recognize the methyl group in m^6^A, one of the most abundant RNA modifications in both plants and animals (F. D. Li *et al*., 2014; Luo and Tong, 2014; Theler *et al.*, 2014; Zhu *et al.*, 2014). Proteins with the YTH domains have in their structure a hydrophobic pocket, usually containing two to three aromatic amino acid residues, essential for m^6^A recognition and binding (F. D. Li *et al.*, 2014; Luo and Tong, 2014; Theler *et al.*, 2014; Zhu *et al.*, 2014). The development of novel sequencing technologies allowed the identification of several YTH domain-encoding proteins in the Arabidopsis genome (D. Y. Li *et al.*, 2014). Most of them belong to the EVOLUTIONARILY CONSERVED C-TERMINAL REGION protein family (ECT1-11) (Ok *et al.*, 2005; D. Y. Li *et al.*, 2014), but, as mentioned above, afunctional domain responsible for m^6^A recognition and binding YTH was also found in the CPSF30-L polyadenylation protein (Delaney *et al.*, 2006; Hou *et al.*, 2021; Song *et al.*, 2021). The connection of m^6^A RNA modification with polyadenylation and APA has been analyzed (Wei *et al.*, 2018; Pontier *et al.*, 2019; Parker *et al.*, 2020), although the experimental evidence for such an interaction was hard to find. In a 2020 study, Parker and colleagues used Nanopore direct RNA sequencing (DRS) analyses to show that both wild-type plants and Arabidopsis mutants (*vir-1*) with compromised m^6^A deposition mechanisms exhibit the disrupted m^6^A pattern. This observation was associated with altered patterns of mRNA cleavage and polyadenylation (Parker *et al*., 2020). At the same time, it has been revealed that m^6^A sites often overlap with the canonical poly(A) signal sequence AAUAAA, and that m^6^A inhibits the polyadenylation complex by selecting downstream proximal cleavage and polyadenylation sites in Arabidopsis (Parker *et al.*, 2020).

In addition to that, Hou and colleagues showed that some Arabidopsis genes (related to nitrate signaling) that have m^6^A modifications in their transcripts are indeed prone to CPSF30-L-mediated alternative polyadenylation regulation (Hou *et al.*, 2021). The choice of poly(A) site is globally regulated by CPSF30-L, which binds m^6^A within FUE (Far Upstream Element) motifs (Song *et al.*, 2021) that are one of the regulatory sequences of polyadenylation (Loke *et al.*, 2005). Interestingly, the YTH domain of this protein is also required for floral transition and abscisic acid (ABA) response in Arabidopsis (Song *et al.*, 2021).

It seems that not only m^6^A modification is connected with poly(A) tail structure. Uridylation has been found to repair deadenylated mRNAs in plants, thus preventing degradation (Zuber *et al.*, 2016). In another Arabidopsis study, a model was proposed in which URT1 nucleotidyl transferase has a dual function, both supporting deadenylated mRNA turnover and preventing excessive deadenylation (Scheer *et al.*, 2021).

## mRNA nuclear export and long-distance transport of modified RNA molecules

RNA modifications are also connected to mRNA nuclear export and long-distance transport. The ALYREF (Aly/REF export factor) protein, which functions as an mRNA transport adaptor during export, can specifically recognize and bind 5ʹ-methylcytosine modified nucleotides (m^5^C) in animal mRNAs (X. Yang *et al*., 2017). This modification was also proven to promote nuclear export of mRNA itself, with the process being coordinated by the m^5^C methyltransferase enzyme NSUN2 (NOP/SUN protein 2) and the ALYFEF protein (X. Yang *et al.*, 2017).

5ʹ-Methylcytosine is also involved in mRNA transport in plants, where transcripts are often moved via phloem between cells or even organs through graft junctions (Yang *et al.*, 2019). Arabidopsis *dmnt2 nsun2b* double mutants, characterized by inactive m^5^C methylases, showed a lack of mobility of two transcripts, *TCTP1* (translationally controlled tumor protein 1) and *HSC70.1* (heat shock cognate protein 70.1), which were confirmed to be successfully transported in wild-type plants (Yang *et al.*, 2019).

## The influence of RNA modifications on translation and mRNA stability

RNA modifications have been widely studied in the context of regulating plant response to stress conditions, when modified nucleotides are involved in regulation of transcript abundance (by modulating mRNA stability or translation) in either direct or indirect ways (Anderson *et al.*, 2018; G. Liu *et al.*, 2020; Cheng *et al.*, 2021; Y. He *et al*., 2021; Hu *et al.*, 2021; Mao *et al.*, 2021; D. Yang *et al.*, 2021; K. Zhang et al., 2021; T. Y. Zhang *et al*., 2021; Hou *et al.*, 2022; Y. Wang *et al*., 2022).For example, m^5^C modification seems to be important for translation efficiency. In rice, methylated cytidine in heat-induced transcripts promoted their translation in plants subjected to high temperature stress (Tang *et al.*, 2020). Mutant plants with inactive OsNSUN2 methylase grown at 36 °C presented strong heat sensitivity phenotypes that were not observed in wild-type plants. This observation showed that m^5^C modification is important for high-temperature acclimation (Tang *et al.*, 2020). Proteomic analyses, ribosome profiling experiments, and luciferase assays, performed by Tang and colleagues, all hinted at higher translation efficiency of transcripts methylated by OsNSUN2, suggesting that m^5^C in mRNA is involved in translational control mechanisms (Tang *et al.*, 2020).

*N* ^6^-Methyladenosine is also connected to translatability and stability of plant mRNAs. The absence of m^6^A marks on Arabidopsis transcripts results in a significant reduction in their abundance (Anderson *et al.*, 2018). The modification was suggested to stabilize transcripts by inhibiting ribonucleolytic cleavage that otherwise takes place directly at the 5ʹ site of methylated adenosine (Anderson *et al.*, 2018). In separate studies, it has been shown that m^6^A lightens intramolecular base pairing in some transcripts, decreasing the loss of mRNA secondary structure (Liu *et al.*, 2015; Spitale *et al.*, 2015; Kramer *et al.*, 2020). This also plays a role in the plant salt stress response (Anderson *et al.*, 2018; Kramer *et al.*, 2020). It was proposed that during salt stress, transcripts encoding stress response proteins are methylated to increase their stability by affecting mRNA secondary structure (Anderson *et al.*, 2018; Kramer *et al.*, 2020). On the other hand, in the Arabidopsis mutants with inactive FIONA1 methyltransferase, the lack of m^6^A marks on PIF4 (PHYTOCHROME INTERACTING FACTOR 4) transcripts resulted in their increased stability(C. L. Wang *et al*., 2022), although it is worth mentioning that the pool of FIONA1-methylated mRNAs is rather small or even non-existent (Parker *et al.*, 2022). In addition, the ECT2 protein (m^6^A reader) was shown to affect trichome morphology in Arabidopsis by affecting mRNA stability in the cytoplasm (Wei *et al.*, 2018). In another study, methylation of adenosines in the 3ʹ-untranslated region (UTR) was shown to regulate expression levels of a pool of salt stress response genes in Arabidopsis by affecting the stability of transcripts (Hu *et al.*, 2021). The modification was also connected to the stability of several transcripts of salt stress negative regulators (Hu *et al.*, 2021). Apart from the stress response, it is also possible that a few plant m^6^A readers, namely ECT2, ECT3, and ECT4, may somehow increase the level of their mRNA targets, either by a direct stabilization of transcripts or by a different, indirect, mechanism (Arribaz-Hernández *et al*., 2021).

*N* ^1^-Methyladenosine (m^1^A) is another RNA modification correlated with translation. The modification was shown to be located near the translation start site of transcripts and their first splice site, as well as in highly structured 5ʹUTRs (Li *et al.*, 2017). The role of m^1^A was also investigated in plants. Mapping of m^1^A in *Petunia hybrida* showed that this modification is abundant in mRNA molecules. It is preferentially located in coding (CDS) regions, closely after the start codon, suggesting its potential role in translation, although this needs further investigation (Yang *et al.*, 2020).

## Regulation of miRNA biogenesis by RNA modification

Plant miRNAs are short (usually 20–24 nt long), non-coding RNAs transcribed by RNAPII and encoded by *MIR* genes, hundreds of which can be found in each plant genome (Kozomara *et al.*, 2019). Primary transcripts of *MIR* genes, known as pri-miRNAs, can reach thousands of nucleotides in length and, like mRNAs, are modified by the addition of a 5ʹ-cap and a 3ʹ-poly(A) tail (reviewed in Bajczyk *et al.*, 2023). They undergo a complicated, multi-step maturation process, led by a microprocessor complex, consisting of DCL1 (DICER LIKE1), HYL1 (HYPONASTIC LEAVES 1), and SE (SERRATE) proteins (Fang and Spector, 2007; Dong *et al.*, 2008). Another protein, HEN1 (HUA ENHANCER 1), modifies both strands of RNA created by microprocessor complex miRNA/miRNA* duplexes, by the addition of a 2ʹ-*O*-methyl group to their 3ʹ-terminal nucleotides (Li *et al.*, 2005; Yu *et al.*, 2005; Baranauske *et al.*, 2015). In Arabidopsis *hen1* null mutants, the level of small RNAs is low, and miRNAs (as well as siRNAs) are simultaneously 3ʹ truncated and 3ʹ uridylated (Li *et al.*, 2005). This shows that 2ʹ-*O*-methylation protects plant miRNAs from 3ʹ–5ʹ truncation and degradation (Li *et al.*, 2005), and this process is now considered to be the main regulator of miRNA stability (reviewed in Zhang *et al.*, 2022). Later, it was found that in the *hen1* mutant (in which miRNA cannot be methylated), most of the miRNAs were uridylated by the HESO1 (HEN1 SUPPRESSOR 1) protein and URT1 nucleotidyl transferase (Park *et al.*, 2002; Ren *et al.*, 2012; Zhao *et al.*, 2012; Tu *et al.*, 2015). Introduction of a loss-of-function mutation of *HESO1* in the *hen1* Arabidopsis mutant background leads to the restoration of miRNA abundance in plants (Ren *et al.*, 2012).

Recently, it was shown that m^6^A modification is also present in pri-miRNAs, and it affects their structure and processing into mature miRNAs (Bhat *et al.*, 2020). Pri-miRNAs were demonstrated to be methylated by MTA, the Arabidopsis enzyme that is homologous to human METTL3 (the catalytic subunit of the human RNA methylation writer complex) (Bhat *et al.*, 2020). Levels of a subset of mature miRNAs were decreased in *A. thaliana* mutants with lower levels of active MTA. This observation leads to the suggestion that MTA is involved in the regulation of biogenesis of some miRNAs at the initial stages of this multi-step process (Bhat *et al.*, 2020). This model was supported by the finding that MTA interacts directly with RNAPII and TOUGH (TGH is a protein that is involved in the early steps of miRNA biogenesis and participates in HYL1 recruitment) (Ren *et al.*, 2012; Bhat *et al.*, 2020).

Apart from the role of m^6^A in plant miRNA biogenesis, it was found that in soybean, 21 nt long miR1510 is only partially 2ʹ-*O*-methylated at the 3ʹ terminus (Fei *et al.*, 2018). It was suggested that an unusual secondary structure of these miRNA precursors leads to the formation of a miR1510/miR1510* duplex with a mismatch adjacent to the 2 nt long 3ʹ overhang. The mismatch inhibits HEN1 methylation activity and subsequently leads to monouridylation by the HESO1 uridyltransferase, resulting in the creation of 22 nt miRNA that later triggers the production of phased secondary siRNA (phasiRNA) molecules (Fei *et al.*, 2013, 2018).

There are several differences between plant and animal miRNA biogenesis pathways including: gene organization, processing steps, proteins involved in the pathway, and the way in which mature miRNA functions. The effects of RNA modifications on animal miRNA biogenesis and function have been widely described. For example, m^6^A has been shown to affect the cleavage of pre-miRNA by Dicer enzyme, which leads to the promotion of miRNA maturation (Alarcon *et al.*, 2015; H. Wang *et al*., 2019). In addition, monouridylation can enhance Dicer processing of animal pre-miRNAs, which have been proven for let-7 and miR-105 precursors, and may protect the molecules from 3ʹ exonucleases (Heo *et al.*, 2012; Kim *et al.*, 2020). Animal pseudouridine synthases, namely PUS10 and TruB1, have been shown to promote and enhance maturation of miRNA precursors (Kurimoto *et al.*, 2020; Song *et al.*, 2020). Finally, inosine can also be found in both mammalian mature miRNAs and their precursors (Luciano *et al.*, 2004; Blow *et al.*, 2006; Yang *et al.*, 2006). A-to-I editing is led by ADAR (adenosine deaminase that acts on RNA) enzymes. It was shown that the binding of ADARs to human miRNA precursors not only catalyzes the conversion of adenosine to inosine but also blocks processing events led by both Drosha and Dicer proteins, interfering with miRNA biogenesis (Ekdahl *et al.*, 2012). This modification was also proven to influence miRNA function, as the increased inosine levels on miR-381 and miR-376b influenced their target recognition (Ekdahl *et al.*, 2012). However, the question of whether inosine and pseudouridine have any role in the biogenesis of plant miRNAs still needs to be answered. The above-mentioned findings on the role of RNA modifications in miRNA biogenesis in animals should guide further research in plants, as the topic is certainly worth exploring.

## Conclusions and perspectives

**Fig. 1.**
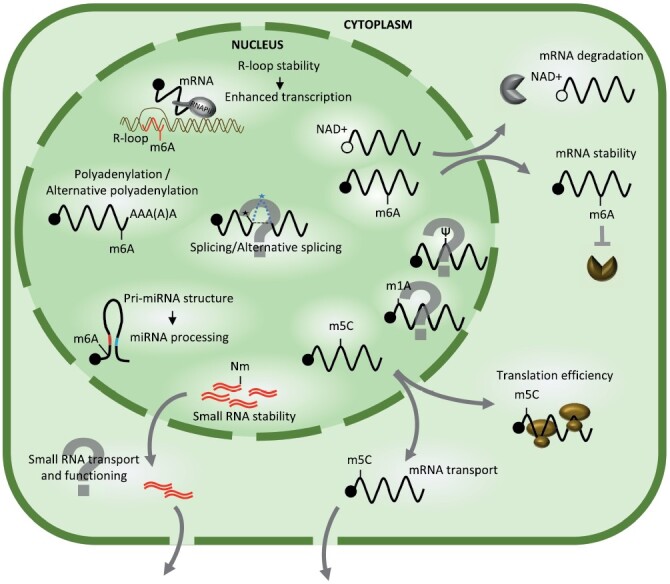
RNA modifications are important for the regulation of gene expression in plants. Modified ribonucleotides affect every stage of RNA life, starting from transcription, through maturation, transport, stability, and translation. Epitranscriptomic marks are prevalent in mRNAs and RNAPII-derived non-coding RNAs, including *MIR* gene transcripts. For plants, the role of some RNA modifications still needs to be elucidated (marked in the figure by question marks).

In recent years, the number of reports pointing to the key role of RNA modifications has rapidly grown. This trend is particularly noticeable for mRNAs. Modified nucleotides provide another stage that could impact the final product of genetic information—the level of functional protein. By inducing or repressing transcription and affecting the stability or maturation of the transcript, RNA modifications can shape the proteome and, as a consequence, change the fate of the cell (Fig. 1). This is true for all living organisms; however, our knowledge of the plant epitranscriptome is still very limited. Currently, many plant scientists are exploring this field to find mechanisms similar to those described already for animals or those that are unique for plants. For many RNA modifications, the enzymes introducing them are not known, or the specificity of the known writers is not yet described; for example, in Arabidopsis, there are 20 pseudouridine synthases identified, but their functions and targets are predicted only based on their putative subcellular localization (Xie *et al.*, 2022). We still do not know enough about readers and erasers of epitranscriptomic marks, and this is why much effort must be made to identify proteins specifically recognizing modified nucleotides. We can imagine that these molecular marks can determine RNA fate, not only how it will be processed but also if it will be exported or retained in the nucleus. Interestingly, in addition to protein-coding RNAs and highly abundant non-coding RNAs (rRNA, tRNA, snRNA, and snoRNA), other non-coding RNAs (e.g. miRNA and siRNA) can also be modified. Today, for plants, we know that 2ʹ-*O*-methylation is important for miRNA stability (Ren *et al.*, 2012; Zhao *et al.*, 2012), and m^6^A, present in some miRNA precursors, is required for their proper structure and processing (Bhat *et al.*, 2020) (Fig. 1). It is almost certain that other modifications are important for the biogenesis and functioning of small RNAs. Recently, several publications have described the extracellular movement of small RNAs (Cai *et al.*, 2018; B. He *et al.*, 2021). We also know that pools of small RNAs are crucial for epigenetic reprogramming and inheritance (Slotkin *et al.*, 2009; Martinez *et al.*, 2016; Long *et al.*, 2021). It would be extremely interesting to find that the modification of small RNAs can decide whether they are transferred from the nucleus to the cytoplasm in vegetative tissues, intracellularly in gametes, or even outside the cell.
